# Superconductivity and Magnetism in Two-Dimensional and Layered Materials

**DOI:** 10.3390/nano15161284

**Published:** 2025-08-21

**Authors:** Erik Piatti

**Affiliations:** Department of Applied Science and Technology, Politecnico di Torino, I-10129 Torino, Italy; erik.piatti@polito.it

In the last twenty years, two-dimensional and layered materials have emerged as a class of compounds that has attracted unprecedented attention from the scientific community. Most of the research efforts have so far been devoted to the exploration of their unique electronic, optical, and optoelectronic properties, also in light of potential technological applications [1,2,3,4,5,6].

More exotic quantum phases have also been discovered in this class of materials, including superconductivity [7,8,9,10,11] and various types of magnetic orders [12,13,14,15]. Several of these fascinating phenomena still elude comprehensive understanding, and new two-dimensional and layered superconducting and/or magnetic compounds are continuously being discovered. As a consequence, the field is in need of novel experimental and theoretical investigations on a fundamental level. Additionally, a subset of these materials is close to attaining technological maturity, which will in turn pave the way for their usage in industry-grade applications with a foreseen impact in different fields, including energy storage [16,17], quantum computing and sensing [18,19], spintronics [20,21], and more.

This Special Issue brings together five research articles dedicated to the exploration of a diverse range of superconducting and magnetic two-dimensional materials. Namely, this Special Issue includes the following: the enhancement of the superconducting properties of polycristalline cuprate superconductor B(P)SCCO through the inclusion of GaN p-n junction luminescent particles [22]; the development of an analytical model for the onset of Faraday–Kerr optical rotation in a wide energy spectrum of single-layer transition metal dichalcogenides upon optical pumping with circularly polarized light [23]; the theoretical modeling of the longitudinal plasma mode occurring in layered cuprate superconductors at large momenta and its signature as an absorption peak in the in-plane optical conductivity when light propagates at small tilting angles relative to the stacking direction [24]; the evolution of the superconducting properties of CaKFe_4_As_4_ single crystals upon electron doping via Co substitution, including the determination of the structure of the energy gap and of the superfluid density up to doping levels hosting the spin–vortex–crystal magnetic order [25]; and the exploration via ab initio simulations of rare-earth ion deposition on the graphene/Ni(111) system as a pathway to induce spin-polarized states in graphene, including the identification of those ions able to provide the required doping and of the mechanisms responsible for charge transfer [26].

This Special Issue aims to promote and accelerate the experimental, theoretical, and computational exploration of superconducting and magnetic two-dimensional and layered materials. It will be of interest to all researchers working on this fascinating class of materials and will attract the attention of the diverse readership of *Nanomaterials*.
